# The identification of high-performing antibodies for Coiled-coil-helix-coiled-coil-helix domain containing protein 10 (CHCHD10) for use in Western Blot, immunoprecipitation and immunofluorescence

**DOI:** 10.12688/f1000research.133479.2

**Published:** 2023-07-26

**Authors:** Riham Ayoubi, Walaa Alshafie, Kathleen Southern, Peter S. McPherson, Carl Laflamme, NeuroSGC/YCharOS/EDDU collaborative group

**Affiliations:** 1 Department of Neurology and Neurosurgery, Structural Genomics Consortium, The Montreal Neurological Institute, McGill University, Montreal, Québec, H3A 2B4, Canada

**Keywords:** Uniprot ID Q8WYQ3, CHCHD10, Coiled-coil-helix-coiled-coil-helix domain-containing protein 10, antibody characterization, antibody validation, Western Blot, immunoprecipitation, immunofluorescence

## Abstract

CHCHD10 is a mitochondrial protein, implicated in the regulation of mitochondrial morphology and cristae structure, as well as the maintenance of mitochondrial DNA integrity. Recently discovered to be associated with amyotrophic lateral sclerosis (ALS) and frontotemporal dementia (FTD) in its mutant form, the scientific community would benefit from the availability of validated anti-CHCHD10 antibodies. In this study, we characterized four CHCHD10 commercial antibodies for Western Blot, immunoprecipitation, and immunofluorescence using a standardized experimental protocol based on comparing read-outs in knockout cell lines and isogenic parental controls. As this study highlights high-performing antibodies for CHCHD10, we encourage readers to use it as a guide to select the most appropriate antibody for their specific needs.

## Introduction

Coiled-coil-helix-coiled-coil-helix domain containing protein 10 (CHCHD10) is a protein localized to the mitochondrial intermembrane space, and is postulated to be involved in the maintenance of mitochondrial organization and cristae structure. ^1^ Twin CX _9_C motifs make up the single coiled-coil-helix-coiled-coil-helix domain, which stabilizes the helix-turn-helix fold. ^2^

Recent studies have demonstrated that CHCHD10 is important for neuronal health. ^1^ As such, *CHCHD10* gene variants have been reported in patients with ALS, FTD, Parkinson’s disease, motor neuron disease, and mitochondrial myopathy, suggesting that they contribute to neurodegenerative disease progression. ^1^^,^^3^^,^^4^ Additional work is needed to understand the underlying function and regulation of CHCHD10, in its native and mutant conformation, to advance the development of therapeutic strategies for targeting these deteriorating diseases. Mechanistic studies would be greatly facilitated with the availability of high-quality CHCHD10 antibodies.

Here, we compared the performance of a range of commercially available CHCHD10 antibodies for Western Blot, immunoprecipitation and immunofluorescence, enabling biochemical and cellular assessment of CHCHD10 properties and function.

## Results and discussion

Our standard protocol involves comparing readouts from wild-type and knockout cells. ^5^^–^^9^ The first step was to identify a cell line(s) that expresses sufficient levels of CHCHD10 to generate a measurable signal to noise. To this end, we examined the DepMap transcriptomics databases to identify all cell lines that express the target at levels greater than 2.5 log _2_ (transcripts per million “TPM” +1), which we have found to be a suitable cut-off (Cancer Dependency Map Portal, RRID:SCR_017655). Commercially available HAP1 cells expressed the *CHCHD10* transcript at RNA levels above the average range of cancer cells analyzed. Parental and *CHCHD10* knockout HAP1 cells were obtained from Horizon Discovery. Parental HCT116 cells were obtained from Abcam for immunoprecipitation experiments ( Table 1).

**Table 1.** Summary of the cell lines used.

Institution	Catalog number	RRID (Cellosaurus)	Cell line	Genotype
Horizon Discovery	C631	CVCL_Y019	HAP1	WT
Horizon Discovery	HZGHC005043c003	CVCL_SI77	HAP1	*CHCHD10* KO
Abcam	ab255451	CVCL_0291	HCT116	WT

**Figure 1.**
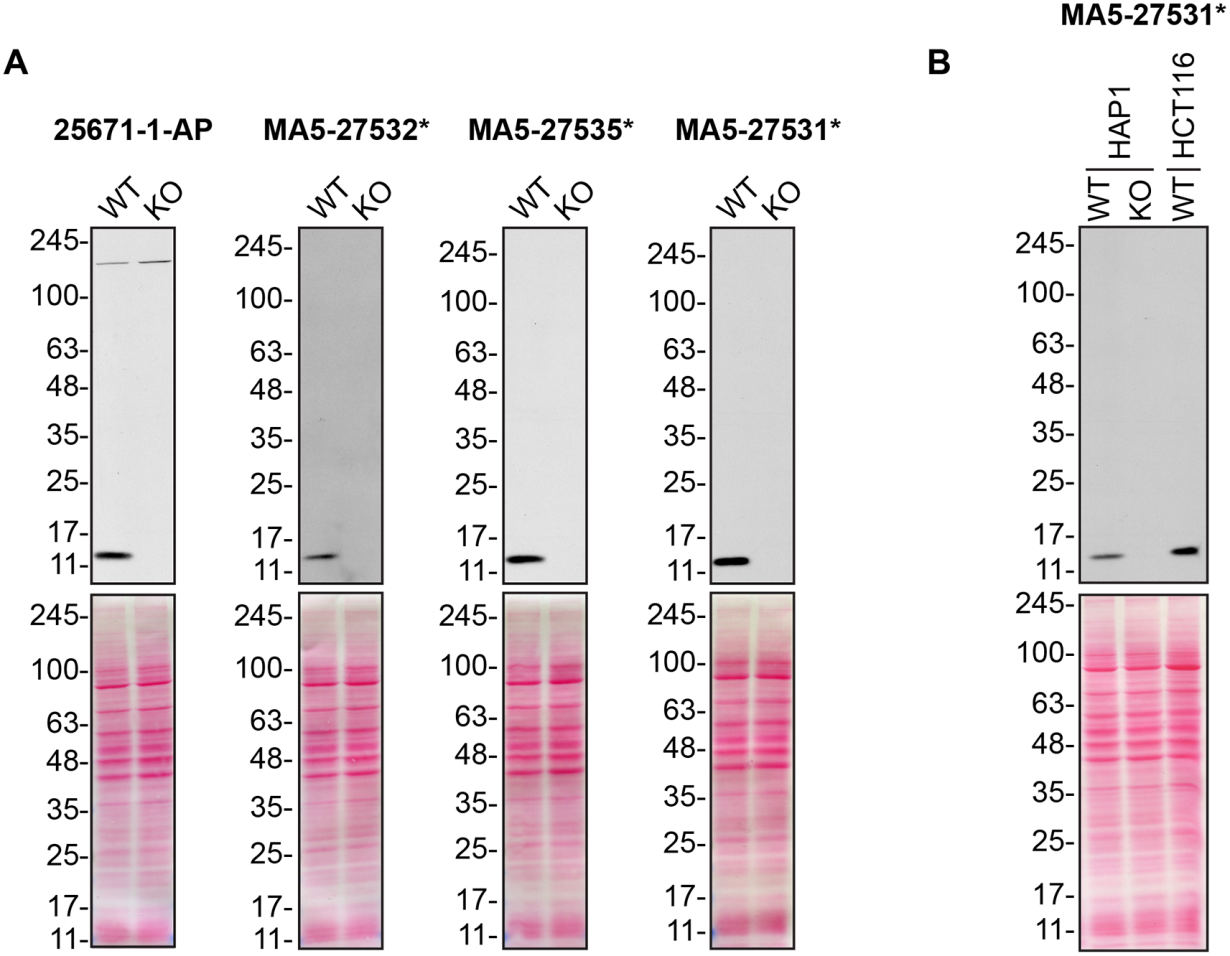
CHCHD10 antibody screening by Western Blot. A) Lysates of HAP1 (WT and *CHCHD10* KO) were prepared, and 50 μg of protein were processed for Western Blot with the indicated CHCHD10 antibodies. The Ponceau stained transfers of each blot are presented to show equal loading of WT and KO lysates and protein transfer efficiency from the polyacrylamide gels to the nitrocellulose membrane. Antibody dilutions were chosen according to the recommendations of the antibody supplier. Antibody dilution used: 25671-1-AP at 1/1000, MA5-27532* at 1/500, MA5-27535* at 1/500, MA5-27531* at 1/500. Predicted band size: 14 kDa. *= monoclonal antibody. B) Lysates of HAP1 (WT and *CHCHD10* KO) and HCT116 were prepared as in A). MA5-27531* was used at 1/500. *= monoclonal antibody.

For Western Blot experiments, we resolved proteins from WT and *CHCHD10* KO cell extracts and probed them side-by-side with all antibodies in parallel ( Figure 1). ^6^^–^^12^

**Figure 2.**
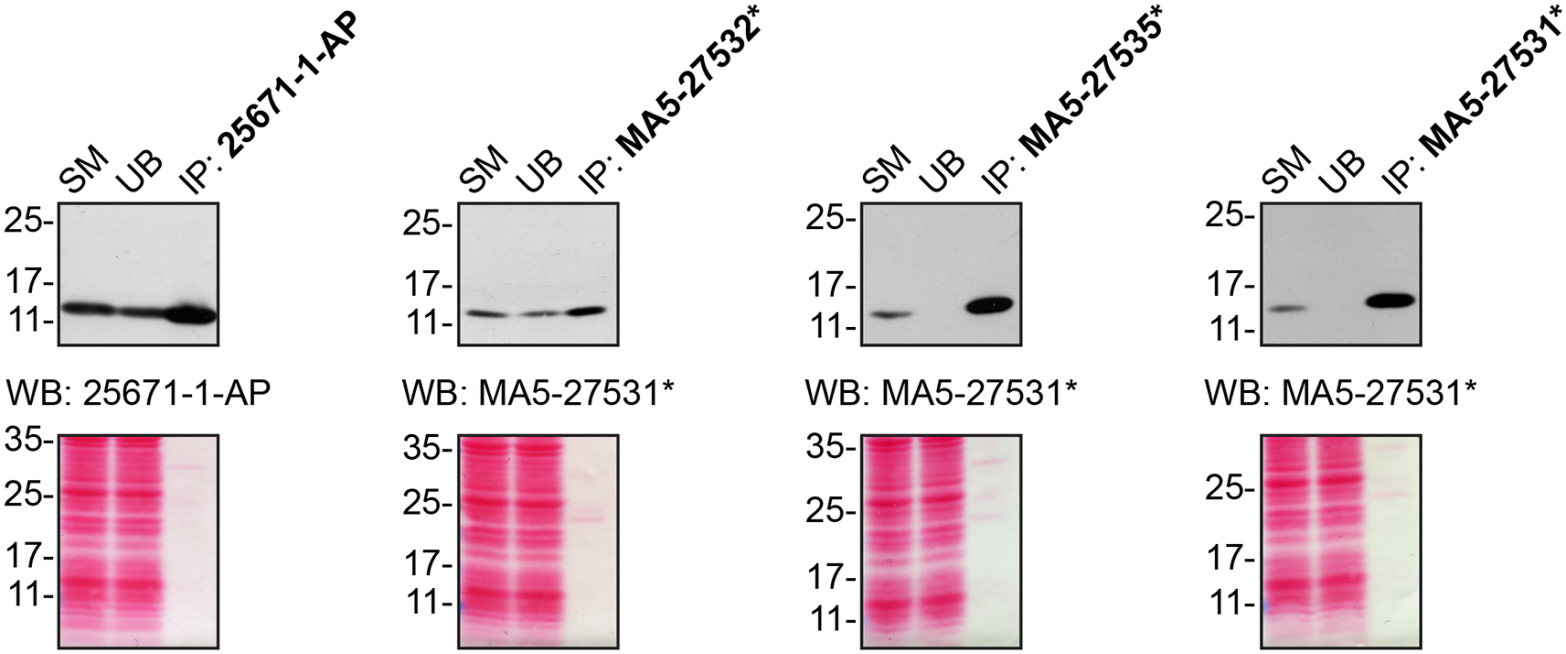
CHCHD10 antibody screening by immunoprecipitation. HCT116 lysates were prepared, and IP was performed using 1.0 μg of the indicated CHCHD10 antibodies pre-coupled to protein A or protein G Sepharose beads. Samples were washed and processed for Western Blot with the indicated CHCHD10 antibody. For Western Blot, 25671-1-AP and MA5-27531* were used at 1/1000. The Ponceau stained transfers of each blot are shown for similar reasons as in Figure 1. SM=10% starting material; UB=10% unbound fraction; IP=immunoprecipitate, *= monoclonal antibody.

For immunoprecipitation experiments, we used the antibodies to immunopurify CHCHD10 from cell extracts. The performance of each antibody was evaluated using Western Blot by detecting the CHCHD10 protein in extracts, in the immunodepleted extracts and in the immunoprecipitates ( Figure 2). ^6^^–^^12^

**Figure 3.**
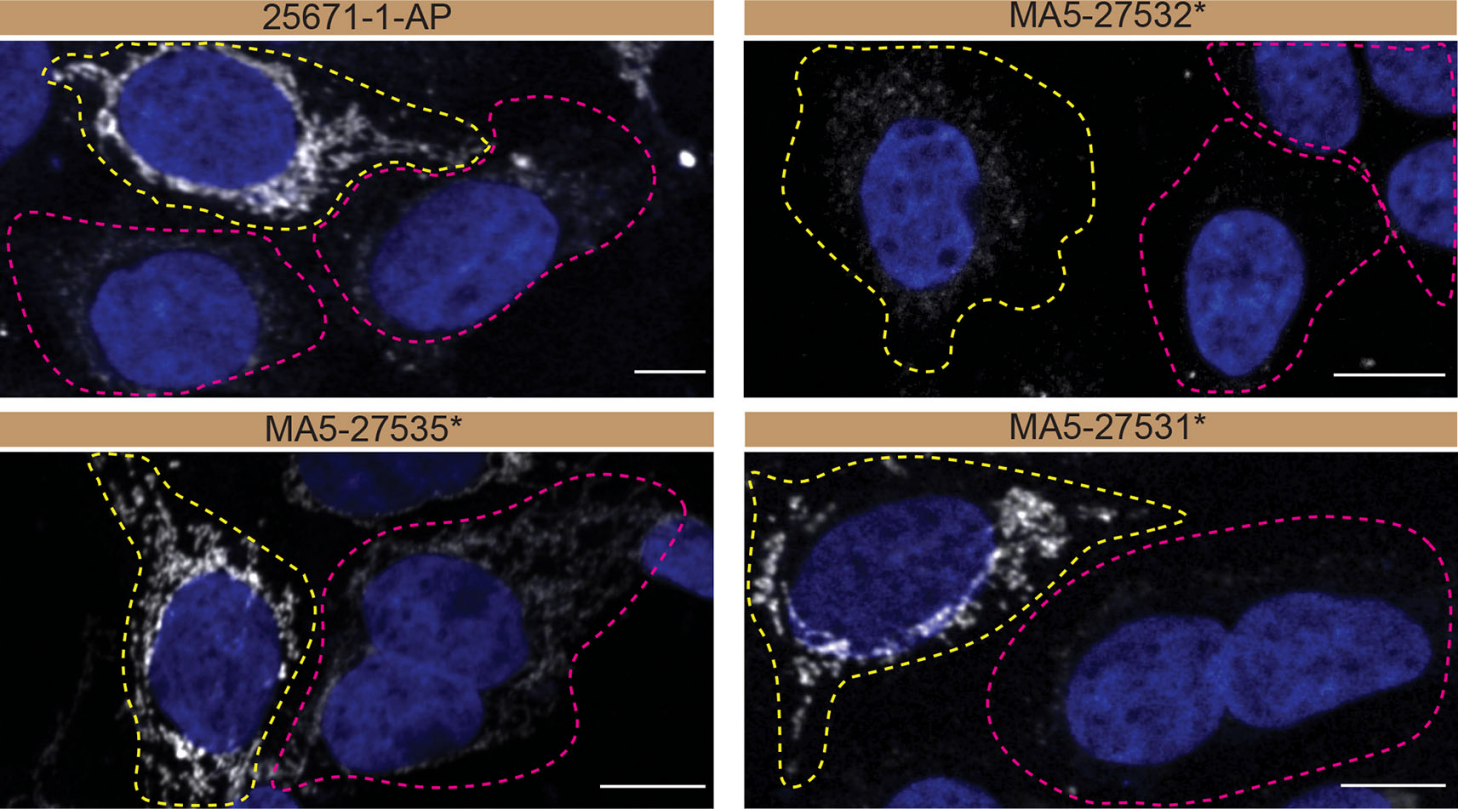
CHCHD10 antibody screening by immunofluorescence. HAP1 WT and *CHCHD10* KO cells were labelled with a green or a far-red fluorescent dye, respectively. WT and KO cells were mixed and plated to a 1:1 ratio on coverslips. Cells were stained with the indicated CHCHD10 antibodies and with the corresponding Alexa-fluor 555 coupled secondary antibody including DAPI. Acquisition of the blue (nucleus-DAPI), green (WT), red (antibody staining) and far-red (KO) channels was performed. Representative images of the merged blue and red (grayscale) channels are shown. WT and KO cells are outlined with yellow and magenta dashed line, respectively. Antibody dilutions were chosen according to the recommendations of the antibody supplier. When the concentration was not indicated by the supplier, which was the case for antibodies MA5-27532* and MA5-27535*, we tested antibodies at 1/100 and 1/1000, respectively. At this concentration, the signal from each antibody was in the range of detection of the microscope used. Antibody dilution used: 25671-1-AP at 1/300, MA5-27532* at 1/100, MA5-27535* at 1/1000, MA5-27531* at 1/100. Bars = 10 μm. *= monoclonal antibody.

For immunofluorescence, as described previously, antibodies were screened using a mosaic strategy. ^13^ In brief, we plated WT and KO cells together in the same well and imaged both cell types in the same field of view to reduce staining, imaging and image analysis bias ( Figure 3).

In conclusion, we have screened CHCHD10 commercial antibodies by Western Blot, immunoprecipitation and immunofluorescence and identified high-quality antibodies under our standardized experimental conditions. The underlying data can be found on Zenodo. ^14^^,^^15^

## Methods

### Antibodies

All CHCHD10 antibodies are listed in Table 2, together with their corresponding Research Resource Identifiers (RRID), to ensure the antibodies are cited properly. ^16^ Peroxidase-conjugated goat anti-rabbit and anti-mouse antibodies are from Thermo Fisher Scientific (cat. number 65-6120 and 62-6520). Alexa-555-conjugated goat anti-rabbit and anti-mouse secondary antibodies are from Thermo Fisher Scientific (cat. number A21429 and A21424).

**Table 2.** Summary of the CHCHD10 antibodies tested.

Company	Catalog number	Lot number	RRID (Antibody Registry)	Clonality	Clone ID	Host	Concentration (μg/μL)	Vendors recommended applications
Proteintech	25671-1-AP	53318	AB_2880187	polyclonal	-	rabbit	0.30	Wb, IP, IF
Thermo Fisher Scientific	MA5-27532 *	VL3152361A	AB_2724131	monoclonal	OTI2B6	mouse	1.00	Wb
Thermo Fisher Scientific	MA5-27535 *	VL3152362A	AB_2724132	monoclonal	OTI3B8	mouse	1.00	Wb
Thermo Fisher Scientific	MA5-27531 *	VL3152369	AB_2724133	monoclonal	OTI4C12	mouse	1.00	Wb

Wb=Western Blot; IF= immunofluorescence; IP=immunoprecipitation.

* = monoclonal antibody.

### Cell culture

Both HAP1 WT and *CHCHD10* KO cell lines used are listed in Table 1, together with their corresponding RRID, to ensure the cell lines are cited properly. ^17^ Cells were cultured in DMEM high-glucose (GE Healthcare cat. number SH30081.01) containing 10% fetal bovine serum (Wisent, cat. number 080450), 2 mM L-glutamate (Wisent cat. number 609065), 100 IU penicillin and 100 μg/mL streptomycin (Wisent cat. number 450201).

### Antibody screening by Western Blot

Western Blots were performed as described in our standard operating procedure. ^18^ HAP1 WT and *CHCHD10* KO were collected in RIPA buffer (50 mM Tris pH 8.0, 150 mM NaCl, 1.0 mM EDTA, 1% Triton X-100, 0.5% sodium deoxycholate, 0.1% SDS) supplemented with 1x protease inhibitor cocktail mix (MilliporeSigma, cat. number 78429). Lysates were sonicated briefly and incubated for 30 min on ice. Lysates were spun at ~110,000 x g for 15 min at 4°C and equal protein aliquots of the supernatants were analyzed by SDS-PAGE and Western Blot. BLUelf prestained protein ladder from GeneDireX (cat. number PM008-0500) was used.

Western Blots were performed with large 8-16% gradient polyacrylamide gels and transferred on nitrocellulose membranes. Proteins on the blots were visualized with Ponceau S staining (Thermo Fisher Scientific, cat. number BP103-10) which is scanned to show together with individual Western Blot. Blots were blocked with 5% milk for 1 hr, and antibodies were incubated overnight at 4°C with 5% bovine serum albumin (BSA) (Wisent, cat. number 800-095) in TBS with 0,1% Tween 20 (TBST) (Cell Signaling Technology, cat. number 9997). Following three washes with TBST, the peroxidase conjugated secondary antibody was incubated at a dilution of ~0.2 μg/mL in TBST with 5% milk for 1 hr at room temperature followed by three washes with TBST. Membranes were incubated with Pierce ECL from Thermo Fisher Scientific (cat. number 32106) prior to detection with the HyBlot CL autoradiography films from Denville (cat. number 1159T41).

### Antibody screening by immunoprecipitation

Immunoprecipitation was performed as described in our standard operating procedure. ^19^ Antibody-bead conjugates were prepared by adding 1.0 μg of antibody to 500 μL of phosphate-buffered saline (PBS) (Wisent, cat. number 311-010-CL) with 0,01% triton X-100 (Thermo Fisher Scientific, cat. number BP151-500) in a 1.5 mL microcentrifuge tube, together with 30 μL of protein A- (for rabbit antibodies) or protein G- (for mouse antibodies) Sepharose beads. Tubes were rocked overnight at 4°C followed by two washes to remove unbound antibodies.

HCT116 WT were collected in HEPES buffer (20 mM HEPES, 100 mM sodium chloride, 1 mM EDTA, 1% Triton X-100, pH 7.4) supplemented with protease inhibitor. Lysates were rocked for 30 min at 4°C and spun at 110,000 x g for 15 min at 4°C. One mL aliquots at 1.0 mg/mL of lysate were incubated with an antibody-bead conjugate for ~2 hrs at 4°C. The unbound fractions were collected, and beads were subsequently washed three times with 1.0 mL of HEPES lysis buffer and processed for SDS-PAGE and Western Blot on 8-16% polyacrylamide gels. As secondary detections systems, the Veriblot for immunoprecipitation detection reagent and the anti-mouse IgG for immunoprecipitation (HRP) from Abcam (cat. number ab131366 and ab131368, respectively) were used.

### Antibody screening by immunofluorescence

Immunofluorescence was performed as described in our standard operating procedure. ^6^^–^^13^ HAP1 WT and *CHCHD10* KO were labelled with a green and a far-red fluorescence dye, respectively. The fluorescent dyes used are from Thermo Fisher Scientific (cat. number C2925 and C34565). The nuclei were labelled with DAPI (Thermo Fisher Scientific, cat. Number D3571) fluorescent stain. WT and KO cells were plated on glass coverslips as a mosaic and incubated for 24 hrs in a cell culture incubator at 37 ^o^C, 5% CO _2_. Cells were fixed in 4% paraformaldehyde (PFA) (Beantown chemical, cat. number 140770-10ml) in PBS for 15 min at room temperature and then washed 3 times with PBS. Cells were permeabilized in PBS with 0,1% Triton X-100 for 10 min at room temperature and blocked with PBS with 5% BSA, 5% goat serum (Gibco, cat. number 16210-064) and 0.01% Triton X-100 for 30 min at room temperature. Cells were incubated with IF buffer (PBS, 5% BSA, 0,01% Triton X-100) containing the primary CHCHD10 antibodies overnight at 4°C. Cells were then washed 3 × 10 min with IF buffer and incubated with corresponding Alexa Fluor 555-conjugated secondary antibodies in IF buffer at a dilution of 1.0 μg/mL for 1 hr at room temperature with DAPI. Cells were washed 3 × 10 min with IF buffer and once with PBS. Coverslips were mounted on a microscopic slide using fluorescence mounting media (DAKO).

Imaging was performed using a Zeiss LSM 880 laser scanning confocal microscope equipped with a Plan-Apo 40x oil objective (NA = 1.40). Analysis was done using the Zen navigation software (Zeiss). All cell images represent a single focal plane. Figures were assembled with Adobe Photoshop (version 24.1.2) to adjust contrast then assembled with Adobe Illustrator (version 27.3.1).

## Data Availability

Zenodo: Antibody Characterization Report for CHCHD10, https://doi.org/10.5281/zenodo.5259992. ^14^ Zenodo: Dataset for the CHCHD10 antibody screening study, https://doi.org/10.5281/zenodo.7779321. ^15^ Data are available under the terms of the Creative Commons Attribution 4.0 International license (CC-BY 4.0).
