# Supplementary material for: The identification of high-performing antibodies for Coiled-coil-helix-coiled-coil-helix domain containing protein 10 (CHCHD10) for use in Western Blot, immunoprecipitation and immunofluorescence
Source: F1000Res. 2023 Jul 26;12:403. Originally published 2023 Apr 14. [Version 2] doi: 10.12688/f1000research.133479.2 (PMC10521100; doi:10.12688/f1000research.133479.2)
Supplement: Peer review history

# Reviewer report: Reviewer response for version 2

**DOI:** 10.5256/f1000research.153618.r188088

**Published:** 2023-09-14

**Authors:** Andrew D. Nguyen

**Affiliations:** 1 Saint Louis University, St. Louis, Missouri, USA

In this Data Note, the authors report the characterization of four commercially available antibodies for CHCHD10 in several different applications. Their approach was methodical, and the results are clear. The manuscript is well written and the data are presented clearly. Overall, I believe this is a valuable resource to the research community.

 I have a few minor suggestions below to improve the manuscript:

- The authors may consider including a brief discussion and/or interpretation of their results.
- For references 13, 14, 18, 19: including additional information (such as indicating Zenodo and providing DOIs) would help readers find these references.

Are sufficient details of methods and materials provided to allow replication by others?

Yes

Is the rationale for creating the dataset(s) clearly described?

Yes

Are the datasets clearly presented in a useable and accessible format?

Yes

Are the protocols appropriate and is the work technically sound?

Yes

Reviewer Expertise:

Frontotemporal dementia, lipid metabolism, antisense oligonucleotides

I confirm that I have read this submission and believe that I have an appropriate level of expertise to confirm that it is of an acceptable scientific standard.

# Reviewer report: Reviewer response for version 2

**DOI:** 10.5256/f1000research.153618.r190842

**Published:** 2023-09-14

**Authors:** Derek P. Narendra

**Affiliations:** 1 National Institute of Neurological Disorders and Stroke, National Institutes of Health, Bethesda, Maryland, USA

The authors have not addressed my concerns.

 Most importantly, they have not addressed why specific antibodies that have been well-validated in prior publications, such as the anti-CHCHD10 antibody developed by Atlas (Sigma, Cat#HPA003440), were not included in their comparison. The referenced antibody was validated using human KO cells and mouse lines in our publications and those of others (see for instance, Burstein *et al.*, 2018 ^1^, Huang *et al.*, 2018 ^2^ and Liu *et al.*, 2020 ^3^).

 It is important that the authors acknowledge that other antibodies not included in their analysis have been previously validated in other publications. Their conclusions about what antibody is optimal is biased by the exclusion of these antibodies in their evaluation.

Are sufficient details of methods and materials provided to allow replication by others?

Yes

Is the rationale for creating the dataset(s) clearly described?

Partly

Are the datasets clearly presented in a useable and accessible format?

Yes

Are the protocols appropriate and is the work technically sound?

Yes

Reviewer Expertise:

neurogenetics, mitochondrial biology

I confirm that I have read this submission and believe that I have an appropriate level of expertise to confirm that it is of an acceptable scientific standard, however I have significant reservations, as outlined above.

## References

[1] : In vitro and in vivo studies of the ALS-FTLD protein CHCHD10 reveal novel mitochondrial topology and protein interactions. *Hum Mol Genet* .2018;27(1) : 10.1093/hmg/ddx397 160-177 10.1093/hmg/ddx397 29112723PMC5886281

[2] : CHCHD2 accumulates in distressed mitochondria and facilitates oligomerization of CHCHD10. *Hum Mol Genet* .2018;27(22) : 10.1093/hmg/ddy270 3881-3900 10.1093/hmg/ddy270 30084972PMC6216204

[3] : Loss of CHCHD2 and CHCHD10 activates OMA1 peptidase to disrupt mitochondrial cristae phenocopying patient mutations. *Hum Mol Genet* .2020;29(9) : 10.1093/hmg/ddaa077 1547-1567 10.1093/hmg/ddaa077 32338760PMC7268789

# Reviewer report: Reviewer response for version 1

**DOI:** 10.5256/f1000research.146471.r184169

**Published:** 2023-07-19

**Authors:** Derek P. Narendra

**Affiliations:** 1 National Institute of Neurological Disorders and Stroke, National Institutes of Health, Bethesda, Maryland, USA

In general, the paper was well-written, and the data included are of high quality.

 A major concern is that the choice of antibodies excludes certain well validated commercial antibodies, which may skew the findings to favor those of the author’s commercial partners.

 There are few suggestions that would make the manuscript stronger.

1. The rationale for choosing the commercial antibodies tested is not clear. The four come from only two suppliers. A notable omission is the the HPA003440 Atlas Antibodies / Sigma-Aldrich antibody, which has been used in several publications, including those that validated in KO cell lines and mouse models. It is not clear why this was not chosen in the head-to-head comparisons. How the antibodies tested were chosen should be discussed as well as the omission of several that have appeared in prior publications.
2. In the second sentence of the introduction, the authors should clarify that CHCHD10 has a single CHCH domain, which is composed of two CX9C motifs.
3.  In the first sentence of the second paragraph, stating that CHCHD10 is important for neuronal survival implies that loss of its function results in neuronal death. This is not clear from the literature. CHCHD10 KO appears to be well-tolerated in mammals, and the mutations are thought to cause disease by a toxic gain-of-function (GoF) mechanism. This is perhaps best demonstrated in studies that have compared CHCHD10 KO mice to those with patient mutations knocked in (PMID: 30877432 and 35700042) ^1^^,^^2^. This is also discussed in a recent review (PMID: 37021679). The introduction could be modified to clarify this point.
4. Figure 1. It would be helpful to state the concentration of antibody used. A more informative head-to-head comparison might be to keep the concentration of antibody constant rather or in addition to using the supplier's recommended concentration.
5. In Figure 2 the authors should comment on whether it is possible the antibodies would also co-immunoprecipitate CHCHD2. Ideally this would also be assessed experimentally on CHCHD10 KO cells. We have observed cross-reactivity of antibodies for CHCHD2 and CHCHD10 in immunoprecipitation experiments. This affects the interpretation of results as a binding partner attributed to CHCHD10 may actually be binding CHCHD2.
6. For Figure 3, in addition to the shown images it would be helpful to see those windowed for the low intensity signal in the KO cells. This would allow better assessment of what appears to be a mitochondrial signal with some of the antibodies (e.g., MA5-27535 but maybe others). We have observed some cross-reactivity with its paralog CHCHD2. Whether this could be an explanation for the residual mitochondrial signal should be discussed and perhaps evaluated by assessment of double knockout cells.

Are sufficient details of methods and materials provided to allow replication by others?

Yes

Is the rationale for creating the dataset(s) clearly described?

Partly

Are the datasets clearly presented in a useable and accessible format?

Yes

Are the protocols appropriate and is the work technically sound?

Yes

Reviewer Expertise:

neurogenetics, mitochondrial biology

I confirm that I have read this submission and believe that I have an appropriate level of expertise to confirm that it is of an acceptable scientific standard, however I have significant reservations, as outlined above.

## References

[1] : ALS/FTD mutant CHCHD10 mice reveal a tissue-specific toxic gain-of-function and mitochondrial stress response. *Acta Neuropathol* .2019;138(1) : 10.1007/s00401-019-01989-y 103-121 10.1007/s00401-019-01989-y 30877432PMC6571048

[2] : OMA1 mediates local and global stress responses against protein misfolding in CHCHD10 mitochondrial myopathy. *J Clin Invest* .2022;132(14) : 10.1172/JCI157504 10.1172/JCI157504 35700042PMC9282932

[3] : CHCHD2 and CHCHD10-related neurodegeneration: molecular pathogenesis and the path to precision therapy. *Biochem Soc Trans* .2023;51(2) : 10.1042/BST20221365 797-809 10.1042/BST20221365 37021679

# Reviewer report: Reviewer response for version 1

**DOI:** 10.5256/f1000research.146471.r176514

**Published:** 2023-06-26

**Authors:** Yang Liu

**Affiliations:** 1 Chengdu Institute of Biology, University of the Chinese Academy of Sciences, Beijing, Beijing, China

Thank you for the opportunity to review the manuscript entitled "The identification of high-performing antibodies for Coiled-coil-helix-coiled-coil-helix domain containing protein 10 (CHCHD10) for use in Western Blot, immunoprecipitation and immunofluorescence ."

 Four commercial antibodies for CHCHD10, a mitochondrial protein that affects mitochondrial morphology and DNA integrity, are profiled in the study. Mutations in CHCHD10 have been associated to ALS and FTD. Using a consistent experimental procedure based on comparing read-outs in knockout cell lines and isogenic parental controls, the researchers characterized four CHCHD10 commercial antibodies for Western Blot, immunoprecipitation, and immunofluorescence. The study discovered high-performing antibodies for CHCHD10 and recommends that readers use it as a guide to select the appropriate antibody for their specific needs.

 Overall, I find the paper to be well-written and informative. However, I recommend providing more discussion and interpretation of the results. It is best to evaluate the advantages and disadvantages of the following types of antibodies. Thank you for considering my feedback.

Are sufficient details of methods and materials provided to allow replication by others?

Yes

Is the rationale for creating the dataset(s) clearly described?

Yes

Are the datasets clearly presented in a useable and accessible format?

Yes

Are the protocols appropriate and is the work technically sound?

Yes

Reviewer Expertise:

Molecular mechanisms for infrared sensing in snakes and function & potential medical applications of snake venom.

I confirm that I have read this submission and believe that I have an appropriate level of expertise to confirm that it is of an acceptable scientific standard.
